# Colon cancer-derived oncogenic EGFR G724S mutant identified by whole genome sequence analysis is dependent on asymmetric dimerization and sensitive to cetuximab

**DOI:** 10.1186/1476-4598-13-141

**Published:** 2014-06-04

**Authors:** Jeonghee Cho, Adam J Bass, Michael S Lawrence, Kristian Cibulskis, Ahye Cho, Shi-Nai Lee, Mai Yamauchi, Nikhil Wagle, Panisa Pochanard, Nayoung Kim, Angela KJ Park, Jonghwa Won, Hyung-Suk Hur, Heidi Greulich, Shuji Ogino, Carrie Sougnez, Douglas Voet, Josep Tabernero, Jose Jimenez, Jose Baselga, Stacey B Gabriel, Eric S Lander, Gad Getz, Michael J Eck, Woong-Yang Park, Matthew Meyerson

**Affiliations:** 1 Department of Medical Oncology, Dana-Farber Cancer Institute, Boston MA 02115, USA; 2 Center for Cancer Genome Discovery, Dana-Farber Cancer Institute, Boston MA 02115, USA; 3 Samsung Genome Institute, Samsung Medical Center, Seoul 135-967, Republic of Korea; 4 Samsung Advanced Institute for Health Sciences and Technology, SungKyunKwan University, Seoul 135-967, Republic of Korea; 5 The Broad Institute of MIT and Harvard, Cambridge MA 02142, USA; 6 Oncology team, Mogam Biotechnology Research Institute, Yongin 446-799, Republic of Korea; 7 Department of Medicine, Brigham and Women's Hospital, Harvard Medical School, Boston MA 02115, USA; 8 Molecular Pathology Laboratory, Vall d’Hebron University Hospital, Universitat Autònoma de Barcelona, Barcelona, Spain; 9 Memorial Sloan-Kettering Cancer Center, New York, NY 10065, USA; 10 Department of Cancer Biology, Dana-Farber Cancer Institute, Boston MA 02115, USA; 11 Department of Biological Chemistry and Molecular Pharmacology, Harvard Medical School, Boston MA 02115, USA; 12 Department of Pathology, Harvard Medical School, Boston, MA 02115, USA

## Abstract

**Background:**

Inhibition of the activated epidermal growth factor receptor (EGFR) with either enzymatic kinase inhibitors or anti-EGFR antibodies such as cetuximab, is an effective modality of treatment for multiple human cancers. Enzymatic EGFR inhibitors are effective for lung adenocarcinomas with somatic kinase domain *EGFR* mutations while, paradoxically, anti-EGFR antibodies are more effective in colon and head and neck cancers where *EGFR* mutations occur less frequently. In colorectal cancer, anti-EGFR antibodies are routinely used as second-line therapy of *KRAS* wild-type tumors. However, detailed mechanisms and genomic predictors for pharmacological response to these antibodies in colon cancer remain unclear.

**Findings:**

We describe a case of colorectal adenocarcinoma, which was found to harbor a kinase domain mutation, G724S, in *EGFR* through whole genome sequencing. We show that G724S mutant EGFR is oncogenic and that it differs from classic lung cancer derived EGFR mutants in that it is cetuximab responsive *in vitro*, yet relatively insensitive to small molecule kinase inhibitors. Through biochemical and cellular pharmacologic studies, we have determined that cells harboring the colon cancer-derived G719S and G724S mutants are responsive to cetuximab therapy in vitro and found that the requirement for asymmetric dimerization of these mutant EGFR to promote cellular transformation may explain their greater inhibition by cetuximab than small-molecule kinase inhibitors.

**Conclusion:**

The colon-cancer derived G719S and G724S mutants are oncogenic and sensitive *in vitro* to cetuximab. These data suggest that patients with these mutations may benefit from the use of anti-EGFR antibodies as part of the first-line therapy.

## Findings

Activation of the epidermal growth factor receptor (EGFR) oncoprotein, a member of the ErbB family of receptor tyrosine kinases, is among the most common oncogenic driving events in human cancer [1]. Genomic mechanisms for activating the *EGFR* gene include nucleotide substitutions and in-frame insertions/deletions of the kinase domain in lung adenocarcinoma and papillary thyroid carcinomas, and multi-exonic deletions (exons 2 through 7: EGFR variant III or vIII), nucleotide substitutions of the extracellular domain and carboxyl terminal deletions in glioblastoma [2-6]. *EGFR* is also activated by high-copy amplifications in many epithelial cancer types, prominently in lung and upper gastrointestinal carcinomas as well as glioblastoma and head and neck cancer [7-10]. Furthermore, EGFR protein is over-expressed in many cancers even without evidence of focused genomic alteration, as observed in many cases of colorectal carcinoma where *EGFR* kinase domain mutations were found in only 3 out of 224 cases, 1.3% subjected to whole exome sequencing [11,12]. Given the elevated expression and genomic alterations present in EGFR, multiple cancer therapies have targeted EGFR, as both its kinase activity and its dependence on extracellular ligand signaling have rendered EGFR vulnerable to therapeutic intervention. FDA-approved EGFR targeted inhibitors include the low-molecular-weight ATP-competitive kinase inhibitors, such as gefitinib and erlotinib, and humanized monoclonal antibodies directed against the extracellular domain, notably cetuximab and panitumumab [13]. Although high-level expression of EGFR ligands and/or increased EGFR gene copy numbers may be predictive markers for antitumor response by cetuximab in colon cancer [14-16], and patients with RAS driven cancers are known not to benefit from cetuximab treatment, a clear molecular explanation of cancer response to cetuximab has remained elusive.

## Genomic studies identify G724S mutant in colorectal carcinomas

Colorectal adenocarcinoma has been a classic model to study the progressive accumulation of genomic lesions leading to cellular transformation. Key genomic features of these tumors involve inactivation of tumor suppressors such as *APC, TP53* and *SMAD4* and mutational activation of oncogenes including *KRAS, NRAS, BRAF* and *PIK3CA*[17]. Given the role of cetuximab in therapy of these cancers, initial efforts to identify activating EGFR mutations identified few such events, though potentially activating events such as G719S were seen [18]. More recent reports have also identified potentially mutations of *ERBB3*[19] and amplifications and mutations of *ERBB2* in CRC [12,20]. We have previously reported whole genome sequence analysis of nine colorectal carcinoma/normal pairs,leading to the identification of activating translocations of *TCF7L2* and of the association of *Fusobacterium nucleatum* with colorectal carcinomas [21,22]. Here, we report genomic analysis of a tenth anonymized case of colorectal carcinoma. Whole genome sequencing was performed on the genomic DNA from colorectal carcinoma tissue and adjacent non-neoplastic colonic tissue to a median coverage of 32.5x and 34.2x coverage, respectively, with 86.8% of the genome sequenced to adequate depth for mutation calling.

**Figure 1:**
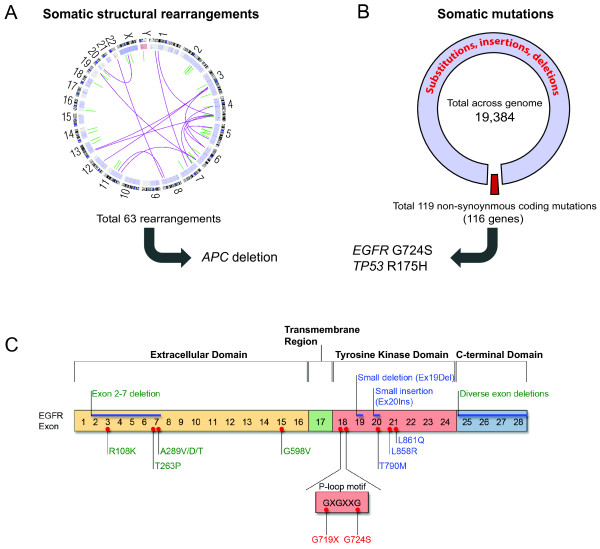
**Identification of a somatic *EGFR *mutation in colorectal adenocarcinoma via whole genome sequencing. (A)** Depiction of the somatic structural rearrangements in this colorectal cancer genome by a Circos plot. The chromosomes are depicted along the circle with somatic rearrangements depicted in purple (interchromosomal) and green (intrachromosomal), including a deletion at the *APC* tumor suppressor locus. **(B)** Depiction of numbers of candidate mutations and non-synonymous alterations in coding genes, and mutations in known cancer genes, *TP53* and *EGFR*. **(C)** Schematic of somatic *EGFR* mutations found in glioblastoma (green lettering), lung adenocarcinoma (blue lettering) and colorectal adenocarcinoma (red lettering), with insertions and deletions above the domain structure, and substitution mutations below the domain structure indicated by red dots.

An analysis of somatic genome structural alterations by comparison of tumor-derived and non-neoplastic derived sequences identified 63 somatic structural rearrangements, including a deletion of the *APC* tumor suppressor gene (Figure 1A, Additional file 1: Figure S1A, and Additional file 2: Table S1). Comparison of nucleotide sequences between the colorectal tumor and normal colon identified an overall mutation rate of 6.7 mutations/Mb including 18,401 somatic nucleotide substitutions, and 983 somatic insertions and deletions of < 37 bases (Figure 1B and Additional file 3: Table S2). As observed in other colorectal cancers [21,23,24], mutation analysis identified a marked elevation in the rate of C to T transitions at CpG dinucleotides (82/Mb). Analysis of non-synonymous coding mutations revealed a total of 119 alterations in 116 genes (Additional file 4: Table S3). Prominent mutations included a somatic R175H substitution in the *TP53* tumor suppressor gene and a somatic G724S substitution in the *EGFR* oncogene (Figure 1B and Additional file 1: Figure S1B). Somatic mutations of common colorectal adenocarcinoma oncogenes *KRAS, BRAF, NRAS* and *PIK3CA*[23] were not detected.

The absence of both *KRAS* and *BRAF* mutations are common features seen in colorectal cancers that are responsive to cetuximab [25,26], thus making the *EGFR* mutation in this case of particular interest. The somatic G724S mutation in *EGFR* occurs at the final glycine of the GxGxxG nucleotide-binding motif that is essential for ATP binding and is conserved among all protein kinases (Figure 1C) [27,28]. Substitution of EGFR G719, the first residue of this motif, to serine, cysteine, or alanine, has been observed in lung adenocarcinomas (~1%), and one G719S mutant and four G724S mutants have been reported in colorectal carcinomas that were sequenced for EGFR (Figure 1C) [18,29] (COSMIC database). In addition, these EGFR mutations were found to be mutually exclusive with well known *KRAS*, *BRAF* and *PIK3CA* oncogenic driver mutations, demonstrating their potential role in tumorigenesis (COSMIC database).

## Colon-cancer derived G719S and G724S mutants are oncogenic and sensitive to cetuximab

To determine whether the G724S mutant is oncogenic and to evaluate its pharmacologic sensitivity, we generated this mutant *in vitro* and retrovirally transduced it into NIH-3T3 cells. While wild-type EGFR expressing NIH-3T3 cells form colonies in soft agar only in the presence of ligand, NIH-3T3 cells that express EGFR G724S form colonies in the absence of exogenous ligand, as do the lung and colon cancer-derived G719S mutants (Additional file 1: Figure S2A) [30]. Furthermore, both G719S and G724S mutants undergo constitutive tyrosine-phosphorylation, which is further increased by EGF treatment, whereas phosphorylation of wild-type EGFR requires induction by EGF (Additional file 1: Figure S2B). These data demonstrate that colon-cancer derived G719S and G724S mutants are oncogenically active in the absence of ligand stimulation.

**Figure 2:**
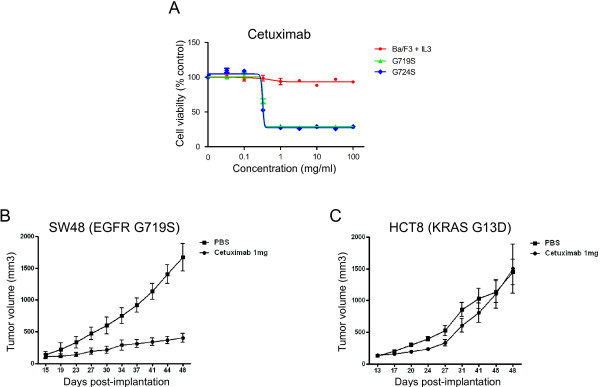
**Pharmacological effects of cetuximab against oncogenic G719S and G724S mutants *in vitro *and *in vivo. *(A)** Cetuximab suppresses the growth of Ba/F3 cells dependent upon the G719S and G724S mutants, but not control cells. Ba/F3 cells transformed with the indicated EGFR mutants were treated with cetuximab at the concentrations indicated and assayed for viability after 72 hours of drug treatment. The results are indicated as mean +/- SD of sextuplicate wells and are representative of three independent experiments **(B** and **C)** Cetuximab is effective against SW48 (EGFR G719S mutant)-induced tumors but not HCT8 (KRAS G13D mutant) induced-tumors in xenografted mice. BALB/c-nu/nu mice (6–8 weeks of age) were injected subcutaneously to the flank with 0.5 ~ 1x10^7^ SW48 or HCT8 cells in 150 ~ 200 μl of PBS. Tumor sizes were measured two times a week using a Vernier caliper and tumor volumes were calculated according to the formula of (short diameter)^2^ x (long diameter)/2. When tumor volume reached around 100 ~ 150 mm^3^, mice were randomized into each group. After confirming that mean tumor volumes were not statistically different between two groups, mice were administered either with PBS or cetuximab (1 mg/mouse) intra-peritoneally twice a week.

To test whether G719S and G724S EGFR mutants are cetuximab-sensitive *in vitro*, we ectopically expressed these mutants in Ba/F3 cells, rendering these cells IL-3 independent but dependent on exogenous oncogenic EGFR signaling [31]. Ba/F3 cells expressing the colon-cancer derived G724S mutant and the lung/colon cancer-derived G719S mutant showed sensitivity to cetuximab with an IC_50_ value of ~ 0.3 μg/ml (Figure 2A). In contrast to their high cetuximab sensitivity, Ba/F3 cells dependent on EGFR G719S and G724S mutants were only moderately sensitive to erlotinib with an IC_50_ value of ~ 0.3 μM (Additional file 1: Figure S3A), consistent with previous reports on G719X mutants *in vitro* and in lung cancer clinical trials [30-33] and with the failure of a previously identified patient with EGFR G724S mutant colorectal cancer to respond to gefitinib [29].

In order to further examine the efficacy of cetuximab, we expanded our studies by generating xenograft mouse models with either SW48 or HCT8 colon cancer cells, which harbor either *EGFR* G719S or *KRAS* G13D mutation, respectively. Here, we found that consistent with *in vitro* response (Additional file 1: Figure S3B), cetuximab treatment dramatically suppressed tumor formation driven by SW48 cells (Figure 2B), suggesting the anti-tumor effect of cetuximab against tumors harboring EGFR G719S mutant. In contrast, cetuximab treatment was ineffective for the tumors driven by HCT8, which is consistent with the previous findings that KRAS-mutant tumors are insensitive to cetuximab (Figure 2C). Taken together, we found that G719S and G724S mutants are oncogenic in the absence of ligand stimulation and effectively respond to cetuximab *in vivo* and *in vitro*.

## Asymmetric dimerization is required for oncogenic activity of G719S and G724S mutant

Recently, we reported that a subset of lung cancer-derived oncogenic EGFR mutants such as L858R require asymmetric dimerization for biochemical activation and oncogenic transforming activity, meaning that their oncogenic ability depend on formation of an EGFR homodimer in which two distinct regions of the two molecule dimerize [34]. By contrast, we showed that other EGFR mutants are oncogenic without the requirement for dimerization, including the gefitinib-resistant exon 20 insertion mutant and the T790M mutant [34]. Furthermore, we found that dimerization-dependent L858R mutant shows a dramatic response to cetuximab, whereas tumors driven by dimerization-independent mutants such as T790M are resistant to the antibody, suggesting that there is a close correlation between dimerization dependency of lung cancer-derived oncogenic mutant EGFR and pharmacological effects of cetuximab [34]. Given that colon cancer-derived G719S and G724S mutants are sensitive to cetuximab, we sought to examine whether oncogenic potential of these mutants are dependent on the asymmetric dimerization like lung cancer-derived L858R mutant.

To test the hypothesis that cetuximab sensitivity of G719S and G724S mutants is a function of their dimerization dependence, we generated G719S and G724S mutants with compound substitution mutations at the dimerization interface in the N-lobe or C-lobe that disrupt the asymmetric dimerization of EGFR [35]. Specifically, we generated epitope-tagged EGFR expression constructs that combined a receiver-impairing mutation (L704N) and/or an activator-impairing mutation (I941R) with oncogenic G719S and G724S mutants. The single or compound EGFR mutants were expressed in NIH-3T3 cells by retroviral transduction, and the EGFR mutant-expressing cells were assayed for their ability to grow in soft agar. In this system, the transforming ability of dimerization-dependent mutants is predicted to be abolished by *cis* mutation of the L704 or I941 mutations. Furthermore, co-expression of the L704N and I941R mutant forms, in contrast, is predicted to restore transforming ability that is dimerization-dependent, because the two mutant forms can heterodimerize. Therefore, this experiment allows us to test whether specific EGFR kinase domain mutants can induce cellular transformation in a dimerization dependent or independent fashion.

**Figure 3:**
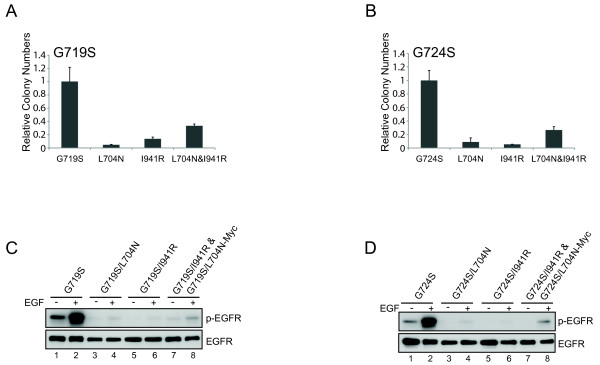
**Dimerization disruption has effects on the transforming activity of G719S and G724S EGFR proteins. (A** and **B)** G719S and G724S mutants are dependent on asymmetric dimerization for their transforming potential. NIH-3T3 cells expressing the indicated EGFR mutants with or without receiver-impairing (L704N) or/and activator-impairing (I941R) mutations were assayed for anchorage-independent growth in soft agar. The bar graph depicts the relative number of colonies in the dimerization-defective mutants normalized to the number of colonies formed by cells expressing the respective parental mutants (n = 3, mean + SD). **(C** and **D)** Ligand-induced and constitutive tyrosine-phosphorylation is abrogated on dimerization-impaired compound mutants of cetuximab-sensitive EGFR mutants. Whole cell lysates from the same cells analyzed in Figure 3A and B expressing G719S **(C)**, and G724S **(D)** mutants with or/and without dimerization-impairing mutations (L704N or I941R) in the absence or presence of EGF treatment for 15 minutes (25 ng/ml) were subjected to immunoblotting with antibodies against phospho-tyrosine (4G10) and EGFR.

Dimerization-impairing *cis* mutations in EGFR, L704N and I941R, significantly reduced the ability of the cetuximab-sensitive G719S and G724S mutants to promote colony formation upon retroviral transduction (Figure 3A and B, L704N or I941R); colony-forming activity was partially or completely restored by co-expression of the L704N and I941R mutants in *trans* (Figure 3A and B, L704N&I941R). Consistent with these results, we found that constitutive and EGF-inducible autophosphorylation of the G719S and G724S mutants (Figures 3C and D, lanes 1 and 2), is attenuated by the introduction of L704N and I941R dimerization-impairing mutations (Figures 3C and D, lanes 3, 4, 5, and 6). Furthermore, receptor phosphorylation is partially rescued by co-expression of either G719S/L704N or G719S/I941R mutants or the cognate combination in a G724S mutant background (Figure 3C and D, lanes 7 and 8). Taken together, these data suggest that colon cancer-derived cetuximab-sensitive G719S and G724S mutants acquire their oncogenic potentials following asymmetric dimerization, respectively, which are similar to lung-cancer derived L858R mutant [35]. Furthermore, these results are consistent with our model that EGFR variants that are dependent on dimerization can be inhibited by cetuximab.

In summary, our findings suggest that *EGFR* mutation may underlie at least some cases of cetuximab responsiveness in colorectal carcinoma. While *EGFR* mutation has historically been believed to be rare in colorectal carcinomas, whole exome sequencing published through The Cancer Genome Atlas identified somatic non-synonymous coding *EGFR* mutations in 10 of 224 colorectal carcinoma cases (or 4.5%) [12]. While many such mutations may be passenger alterations that do not activate EGFR signaling, these results do speak to the potential for mutational activation of EGFR to result in susceptibility to anti-EGFR antibodies in a small fraction of CRC cases. Indeed, the recent demonstration of secondary somatic mutation in the EGFR extracellular domain conferring acquired resistance to cetuximab [36] is consistent with attribution of responses to cetuximab to EGFR blockade. Anti-EGFR therapy in metastatic colorectal cancer has been reserved for second-line therapy after failure of initial empiric chemotherapy but is now increasingly also used as part of first-line therapy for RAS wild-type patients. As genomic diagnostics enter into routine clinical practice, patients whose CRCs harbor potentially oncogenic *EGFR* mutations will be identified, including those at codons 719 and 724. These results suggest that in such patients, therapeutic approaches utilizing EGFR-directed antibody as part of their initial therapy should be evaluated given the greater potential dependence on EGFR signaling in these patients.

## Competing interests

Relating to the content of this work, a patent titled “Methods of predicting response to EGFR antibody therapy” (WO2012065071 A2) was filed by the authors of this manuscript (JB, AB, JC, ML, MM, JT). MM: Inventor on a patent for EGFR mutation analysis in lung cancer, and a consultant to and equity holder in Foundation Medicine. The other authors declare that they have no competing interests.

## Authors’ contributions

JC and AB conceived and designed study; performed experiments; analyzed and interpreted data; wrote the manuscript. ML, KC, NW, CS, DV and GG performed genomic analysis. MY, SO, JT, JJ, JB provided samples and coordinated sample preparation. AC, NK, AP, SL, JW and HH conducted in vivo and in vitro experiments. SG and ESL supervised the genomic analysis process. HG, ME and WP analyzed and interpreted data. MM conceived and designed study; analyzed and interpreted data; wrote the manuscript. All authors read and approved the final manuscript.

## Supplementary Material

Additional file 1 Supplemental Figures. Click here for file

Additional file 2: Table S1 Somatic structural rearrangements identified in a colorectal adenocarcinoma whole genome sequence using the dRanger tool. Click here for file

Additional file 3: Table S2 Somatic mutations and small insertions/deletions identified across the genome of a colorectal adenocarcinoma tumor using whole genome sequencing. Click here for file

Additional file 4: Table S3 Somatic mutations predicted to cause non-synonymous changes in protein coding genes as identified from whole genome sequencing of a colorectal adenocarcinoma. Click here for file

## References

[B1] Hynes NE Lane HA ERBB receptors and cancer: the complexity of targeted inhibitors Nat Rev Cancer 2005 5 341 354 10.1038/nrc1609 15864276

[B2] Chan SK Gullick WJ Hill ME Mutations of the epidermal growth factor receptor in non-small cell lung cancer – search and destroy Eur J Cancer 2006 42 17 23 10.1016/j.ejca.2005.07.031 16364841

[B3] Lynch TJ Bell DW Sordella R Gurubhagavatula S Okimoto RA Brannigan BW Harris PL Haserlat SM Supko JG Haluska FG Louis DN Christiani DC Settleman J Haber DA Activating mutations in the epidermal growth factor receptor underlying responsiveness of non-small-cell lung cancer to gefitinib N Engl J Med 2004 350 2129 2139 10.1056/NEJMoa040938 15118073

[B4] Pao W Miller V Zakowski M Doherty J Politi K Sarkaria I Singh B Heelan R Rusch V Fulton L Mardis E Kupfer D Wilson R Kris M Varmus H EGF receptor gene mutations are common in lung cancers from "never smokers" and are associated with sensitivity of tumors to gefitinib and erlotinib Proc Natl Acad Sci U S A 2004 101 13306 13311 10.1073/pnas.0405220101 15329413 PMC516528

[B5] Paez JG Janne PA Lee JC Tracy S Greulich H Gabriel S Herman P Kaye FJ Lindeman N Boggon TJ Naoki K Sasaki H Fujii Y Eck MJ Sellers WR Johnson BE Meyerson M EGFR mutations in lung cancer: correlation with clinical response to gefitinib therapy Science 2004 304 1497 1500 10.1126/science.1099314 15118125

[B6] Cho J Pastorino S Zeng Q Xu X Johnson W Vandenberg S Verhaak R Cherniack AD Watanabe H Dutt A Kwon J Chao YS Onofrio RC Chiang D Yuza Y Kesari S Meyerson M Glioblastoma-derived epidermal growth factor receptor carboxyl-terminal deletion mutants are transforming and are sensitive to EGFR-directed therapies Cancer Res 2011 71 7587 7596 10.1158/0008-5472.CAN-11-0821 22001862 PMC3242822

[B7] Hirono Y Tsugawa K Fushida S Ninomiya I Yonemura Y Miyazaki I Endou Y Tanaka M Sasaki T Amplification of epidermal growth factor receptor gene and its relationship to survival in human gastric cancer Oncology 1995 52 182 188 10.1159/000227455 7715901

[B8] al-Kasspooles M Moore JH Orringer MB Beer DG Amplification and over-expression of the EGFR and erbB-2 genes in human esophageal adenocarcinomas Int J Cancer 1993 54 213 219 10.1002/ijc.2910540209 8098013

[B9] Leonard JH Kearsley JH Chenevix-Trench G Hayward NK Analysis of gene amplification in head-and-neck squamous-cell carcinoma Int J Cancer 1991 48 511 515 10.1002/ijc.2910480406 2045198

[B10] Cancer Genome Atlas Research N Comprehensive genomic characterization defines human glioblastoma genes and core pathways Nature 2008 455 1061 1068 10.1038/nature07385 18772890 PMC2671642

[B11] Spano JP Lagorce C Atlan D Milano G Domont J Benamouzig R Attar A Benichou J Martin A Morere JF Raphael M Penault-Llorca F Breau JL Fagard R Khayat D Wind P Impact of EGFR expression on colorectal cancer patient prognosis and survival Ann Oncol 2005 16 102 108 10.1093/annonc/mdi006 15598946

[B12] Cancer Genome Atlas N Comprehensive molecular characterization of human colon and rectal cancer Nature 2012 487 330 337 10.1038/nature11252 22810696 PMC3401966

[B13] Robinson KW Sandler AB EGFR tyrosine kinase inhibitors: difference in efficacy and resistance Curr Oncol Rep 2013 15 396 404 10.1007/s11912-013-0323-7 23674236

[B14] Khambata-Ford S Garrett CR Meropol NJ Basik M Harbison CT Wu S Wong TW Huang X Takimoto CH Godwin AK Tan BR Krishnamurthi SS Burris HA 3rd Poplin EA Hidalgo M Baselga J Clark EA Mauro DJ Expression of epiregulin and amphiregulin and K-ras mutation status predict disease control in metastatic colorectal cancer patients treated with cetuximab J Clin Oncol 2007 25 3230 3237 10.1200/JCO.2006.10.5437 17664471

[B15] Yonesaka K Zejnullahu K Lindeman N Homes AJ Jackman DM Zhao F Rogers AM Johnson BE Janne PA Autocrine production of amphiregulin predicts sensitivity to both gefitinib and cetuximab in EGFR wild-type cancers Clin Cancer Res 2008 14 6963 6973 10.1158/1078-0432.CCR-08-0957 18980991 PMC3227691

[B16] Hirsch FR Herbst RS Olsen C Chansky K Crowley J Kelly K Franklin WA Bunn PA Jr Varella-Garcia M Gandara DR Increased EGFR gene copy number detected by fluorescent in situ hybridization predicts outcome in non-small-cell lung cancer patients treated with cetuximab and chemotherapy J Clin Oncol 2008 26 3351 3357 10.1200/JCO.2007.14.0111 18612151 PMC3368372

[B17] Fearon ER Molecular genetics of colorectal cancer Annu Rev Pathol 2011 6 479 507 10.1146/annurev-pathol-011110-130235 21090969

[B18] Barber TD Vogelstein B Kinzler KW Velculescu VE Somatic mutations of EGFR in colorectal cancers and glioblastomas N Engl J Med 2004 351 2883 10.1056/NEJM200412303512724 15625347

[B19] Jaiswal BS Kljavin NM Stawiski EW Chan E Parikh C Durinck S Chaudhuri S Pujara K Guillory J Edgar KA Janakiraman V Scholz RP Bowman KK Lorenzo M Li H Wu J Yuan W Peters BA Kan Z Stinson J Mak M Modrusan Z Eigenbrot C Firestein R Stern HM Rajalingam K Schaefer G Merchant MA Sliwkowski MX de Sauvage FJ Oncogenic ERBB3 mutations in human cancers Cancer Cell 2013 23 603 617 10.1016/j.ccr.2013.04.012 23680147

[B20] Dulak AM Schumacher SE van Lieshout J Imamura Y Fox C Shim B Ramos AH Saksena G Baca SC Baselga J Tabernero J Barretina J Enzinger PC Corso G Roviello F Lin L Bandla S Luketich JD Pennathur A Meyerson M Ogino S Shivdasani RA Beer DG Godfrey TE Beroukhim R Bass AJ Gastrointestinal adenocarcinomas of the esophagus, stomach, and colon exhibit distinct patterns of genome instability and oncogenesis Cancer Res 2012 72 4383 4393 10.1158/0008-5472.CAN-11-3893 22751462 PMC3432726

[B21] Bass AJ Lawrence MS Brace LE Ramos AH Drier Y Cibulskis K Sougnez C Voet D Saksena G Sivachenko A Jing R Parkin M Pugh T Verhaak RG Stransky N Boutin AT Barretina J Solit DB Vakiani E Shao W Mishina Y Warmuth M Jimenez J Chiang DY Signoretti S Kaelin WG Spardy N Hahn WC Hoshida Y Ogino S Genomic sequencing of colorectal adenocarcinomas identifies a recurrent VTI1A-TCF7L2 fusion Nat Genet 2011 43 964 968 10.1038/ng.936 21892161 PMC3802528

[B22] Kostic AD Gevers D Pedamallu CS Michaud M Duke F Earl AM Ojesina AI Jung J Bass AJ Tabernero J Baselga J Liu C Shivdasani RA Ogino S Birren BW Huttenhower C Garrett WS Meyerson M Genomic analysis identifies association of Fusobacterium with colorectal carcinoma Genome Res 2012 22 292 298 10.1101/gr.126573.111 22009990 PMC3266036

[B23] Sjoblom T Jones S Wood LD Parsons DW Lin J Barber TD Mandelker D Leary RJ Ptak J Silliman N Szabo S Buckhaults P Farrell C Meeh P Markowitz SD Willis J Dawson D Willson JK Gazdar AF Hartigan J Wu L Liu C Parmigiani G Park BH Bachman KE Papadopoulos N Vogelstein B Kinzler KW Velculescu VE The consensus coding sequences of human breast and colorectal cancers Science 2006 314 268 274 10.1126/science.1133427 16959974

[B24] Wood LD Parsons DW Jones S Lin J Sjoblom T Leary RJ Shen D Boca SM Barber T Ptak J Silliman N Szabo S Dezso Z Ustyanksky V Nikolskaya T Nikolsky Y Karchin R Wilson PA Kaminker JS Zhang Z Croshaw R Willis J Dawson D Shipitsin M Willson JK Sukumar S Polyak K Park BH Pethiyagoda CL Pant PV The genomic landscapes of human breast and colorectal cancers Science 2007 318 1108 1113 10.1126/science.1145720 17932254

[B25] Karapetis CS Khambata-Ford S Jonker DJ O'Callaghan CJ Tu D Tebbutt NC Simes RJ Chalchal H Shapiro JD Robitaille S Price TJ Shepherd L Au HJ Langer C Moore MJ Zalcberg JR K-ras mutations and benefit from cetuximab in advanced colorectal cancer N Engl J Med 2008 359 1757 1765 10.1056/NEJMoa0804385 18946061

[B26] De Roock W Claes B Bernasconi D De Schutter J Biesmans B Fountzilas G Kalogeras KT Kotoula V Papamichael D Laurent-Puig P Penault-Llorca F Rougier P Vincenzi B Santini D Tonini G Cappuzzo F Frattini M Molinari F Saletti P De Dosso S Martini M Bardelli A Siena S Sartore-Bianchi A Tabernero J Macarulla T Di Fiore F Gangloff AO Ciardiello F Pfeiffer P Effects of KRAS, BRAF, NRAS, and PIK3CA mutations on the efficacy of cetuximab plus chemotherapy in chemotherapy-refractory metastatic colorectal cancer: a retrospective consortium analysis Lancet Oncol 2010 11 753 762 10.1016/S1470-2045(10)70130-3 20619739

[B27] Hanks SK Hunter T Protein kinases 6. The eukaryotic protein kinase superfamily: kinase (catalytic) domain structure and classification FASEB J 1995 9 576 596 7768349

[B28] Hemmer W McGlone M Tsigelny I Taylor SS Role of the glycine triad in the ATP-binding site of cAMP-dependent protein kinase J Biol Chem 1997 272 16946 16954 10.1074/jbc.272.27.16946 9202006

[B29] Ogino S Meyerhardt JA Cantor M Brahmandam M Clark JW Namgyal C Kawasaki T Kinsella K Michelini AL Enzinger PC Kulke MH Ryan DP Loda M Fuchs CS Molecular alterations in tumors and response to combination chemotherapy with gefitinib for advanced colorectal cancer Clin Cancer Res 2005 11 6650 6656 10.1158/1078-0432.CCR-05-0738 16166444

[B30] Greulich H Chen TH Feng W Janne PA Alvarez JV Zappaterra M Bulmer SE Frank DA Hahn WC Sellers WR Meyerson M Oncogenic transformation by inhibitor-sensitive and -resistant EGFR mutants PLoS Med 2005 2 e313 10.1371/journal.pmed.0020313 16187797 PMC1240052

[B31] Jiang J Greulich H Janne PA Sellers WR Meyerson M Griffin JD Epidermal growth factor-independent transformation of Ba/F3 cells with cancer-derived epidermal growth factor receptor mutants induces gefitinib-sensitive cell cycle progression Cancer Res 2005 65 8968 8974 10.1158/0008-5472.CAN-05-1829 16204070

[B32] Han SW Kim TY Hwang PG Jeong S Kim J Choi IS Oh DY Kim JH Kim DW Chung DH Im SA Kim YT Lee JS Heo DS Bang YJ Kim NK Predictive and prognostic impact of epidermal growth factor receptor mutation in non-small-cell lung cancer patients treated with gefitinib J Clin Oncol 2005 23 2493 2501 10.1200/JCO.2005.01.388 15710947

[B33] Sequist LV Besse B Lynch TJ Miller VA Wong KK Gitlitz B Eaton K Zacharchuk C Freyman A Powell C Ananthakrishnan R Quinn S Soria JC Neratinib, an irreversible pan-ErbB receptor tyrosine kinase inhibitor: results of a phase II trial in patients with advanced non-small-cell lung cancer J Clin Oncol 2010 28 3076 3083 10.1200/JCO.2009.27.9414 20479403

[B34] Cho J Chen L Sangji N Okabe T Yonesaka K Francis JM Flavin RJ Johnson W Kwon J Yu S Greulich H Johnson BE Eck MJ Janne PA Wong KK Meyerson M Cetuximab response of lung cancer-derived EGF receptor mutants is associated with asymmetric dimerization Cancer Res 2013 73 6770 6779 10.1158/0008-5472.CAN-13-1145 24063894 PMC3903789

[B35] Zhang X Gureasko J Shen K Cole PA Kuriyan J An allosteric mechanism for activation of the kinase domain of epidermal growth factor receptor Cell 2006 125 1137 1149 10.1016/j.cell.2006.05.013 16777603

[B36] Montagut C Dalmases A Bellosillo B Crespo M Pairet S Iglesias M Salido M Gallen M Marsters S Tsai SP Minoche A Seshagiri S Serrano S Himmelbauer H Bellmunt J Rovira A Settleman J Bosch F Albanell J Identification of a mutation in the extracellular domain of the Epidermal Growth Factor Receptor conferring cetuximab resistance in colorectal cancer Nat Med 2012 18 221 223 10.1038/nm.2609 22270724

