# Supplementary material for: Colon cancer-derived oncogenic EGFR G724S mutant identified by whole genome sequence analysis is dependent on asymmetric dimerization and sensitive to cetuximab
Source: Mol Cancer. 2014 Jun 4;13:141. doi: 10.1186/1476-4598-13-141 (PMC4072491; doi:10.1186/1476-4598-13-141)
Supplement: Additional file 1 — Supplemental Figures. [file 1476-4598-13-141-S1.docx]

**Supplemental Figures**

APC

Read counts across *APC* locus

Normal

Tumor

A


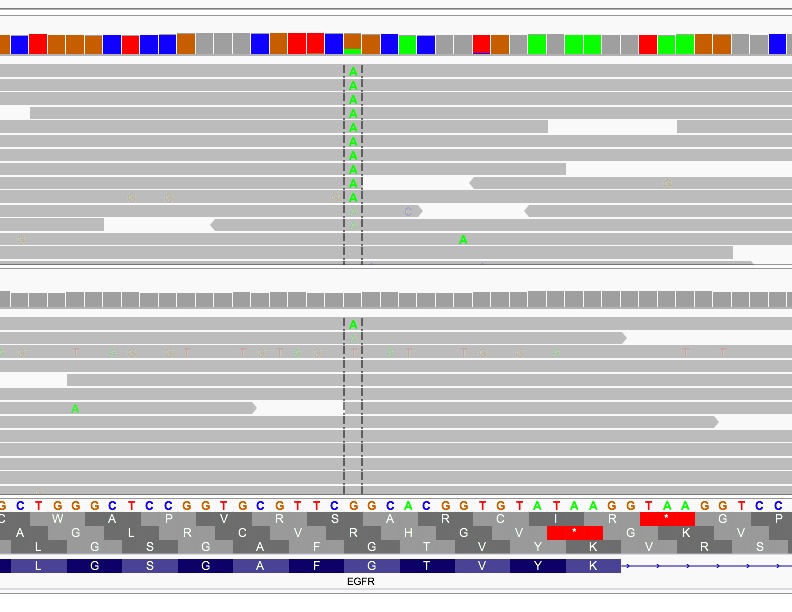

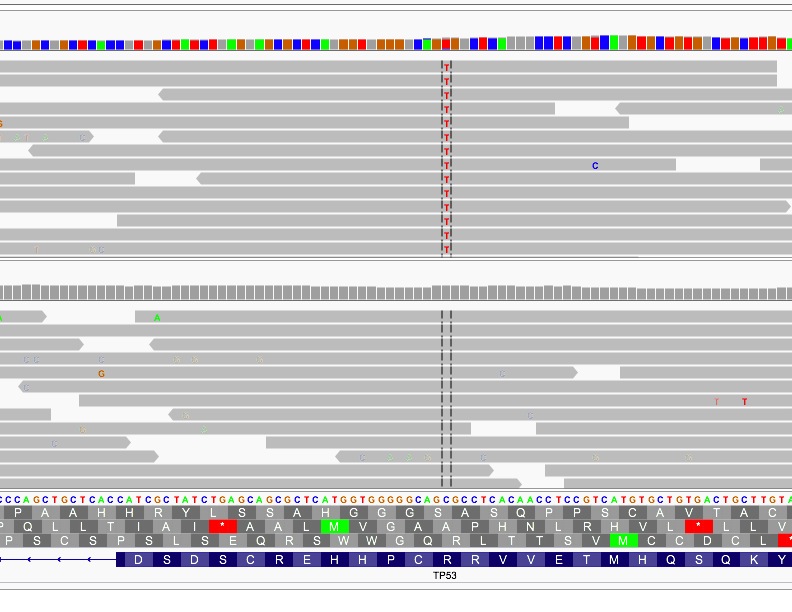


*EGFR* G724S

*TP53* R175H

B

Tumor

Normal

**Figure S1**. Integrative Genome Viewer [[1](#_ENREF_1)] screenshots of (A) somatic *APC* deletion and (B) somatic *EGFR* and *TP53* mutations comparing mutant tumor and wild-type normal sequences. Note the presence of trace tumor-in-normal contamination visible for the *EGFR* mutant

**Figure S2**. Colon cancer-derived G719S and G724S EGFR mutants are oncogenic in the absence of ligands.

(A) NIH-3T3 cells stably expressing G719S, G729S mutants or wild-type EGFR or control cells were subjected to a colony formation assay in soft agar in the absence or presence of EGF. The bar graph shows the number of colonies formed by NIH-T3T3 cells expressing EGFR mutants in soft agar as indicated (n=3, mean +SD). (B) G719S and G724S mutants are constitutively tyrosine-phosphorylated in the absence of EGF and it is further induced by EGF stimulation. The same cells used for the colony formation assay in (A) were subjected to immunoblotting with anti-phospho-tyrosine antibodies (4G10) with or without EGF treatment. Blots were also probed with anti-vinculin antibodies for a loading control.

**References**

1. Thorvaldsdottir H, Robinson JT, Mesirov JP: **Integrative Genomics Viewer (IGV): high-performance genomics data visualization and exploration.** *Brief Bioinform* 2013, **14:**178-192.
